# Humoral Response to Microbial Biomarkers in Rheumatoid Arthritis Patients

**DOI:** 10.3390/jcm10215153

**Published:** 2021-11-02

**Authors:** Seyedesomaye Jasemi, Gian Luca Erre, Maria Luisa Cadoni, Marco Bo, Leonardo A. Sechi

**Affiliations:** 1 Department of Biomedical Sciences, University of Sassari, Viale San Pietro 43b, 07100 Sassari, Italy; s.jasemi@studenti.uniss.it (S.J.); m.bo4@studenti.uniss.it (M.B.); 2 Department of Medical, Surgical and Experimental Sciences, University of Sassari, 07100 Sassari, Italy; glerre@uniss.it (G.L.E.); sardinia@hotmail.com (M.L.C.); 3 Dipartimento di Specialità Mediche, Azienda Ospedaliero Universitaria di Sassari, 07100 Sassari, Italy; 4 Struttura Complessa di Microbiologia e Virologia, Azienda Ospedaliera Universitaria di Sassari, 07100 Sassari, Italy

**Keywords:** rheumatoid arthritis, immune response, *P. gingivalis*, A. *actinomycetemcomitans*, M. avium subspecies *paratuberculosis*, Epstein–Barr virus, human endogenous retroviruses

## Abstract

Background/Objective: Chronic humoral immune response against multiple microbial antigens may play a crucial role in the etiopathogenesis of rheumatoid arthritis (RA). We aimed to assess the prevalence and magnitude of antibody response against various bacterial and viral immunogen peptides in the sera of RA patients compared with the general population. Methods: Polyclonal IgG antibodies (Abs) specific for peptides derived from *Porphyromonas gingivalis* (RgpA, Kpg), *Aggregatibacter *actinomycetemcomitans** (LtxA1, LtxA2), *Mycobacterium avium* subsp. *paratuberculosis* (MAP4027), Epstein–Barr virus (EBNA1, EBVBOLF), and human endogenous retrovirus (HERV-W env-su) were detected by ELISA in serum samples from 148 consecutive RA patients and 148 sex and age-matched healthy controls (HCs). In addition, the presence of a relationship between the positivity and the titer of antibodies and RA descriptors was explored by bivariate correlation analysis. Results: RA patients exhibit a higher prevalence of humoral immune response against all tested peptides compared to HCs with a statically significant difference for MAP4027 (30.4% vs. 10.1%), BOLF (25.7% vs. 8.1%), RgpA (24.3% vs. 9.4%), HERV W-env (20.3% vs. 9.4%), and EBNA1 (18.9% vs. 9.4%) peptides. Fifty-three (35.8%) out of 148 RA serum and 93 (62.8%) out of 148 HCs were negative for all pathogen-derived peptides. There was a significant correlation between OD values obtained by ELISA test against all peptides (*p* < 0.0001). We also found an increased titer and prevalence of Abs against LtxA1 and LtxA2 in seropositive vs. seronegative RF (*p* = 0.019, *p* = 0.018). Conclusion: This study demonstrates a significantly increased humoral response against multiple pathogens in patients with RA and implies that they could be an important factor in the pathogenesis of the disease. Therefore, the role of each individual pathogen in RA needs to be further investigated.

## 1. Introduction

Rheumatoid arthritis (RA) is a chronic systemic autoimmune disorder affecting 0.5–1% of people worldwide [1]. The main clinical manifestation of the disease is the chronic inflammatory process in the joints, which results in bone damage, loss of function, and reduced independence in performing daily activities [2].

The etiology of RA is not well understood. However, several triggers, such as smoking, infections, and microbiota, have been identified as risk factors for initiating and exacerbating the disease in genetically susceptible individuals [3]. Autoantibodies against different self-antigens such as rheumatoid factor (RF) and anti-citrullinated protein antibody (ACPA) are considered immunological markers of RA and can be found years before the onset of overt symptoms [4]. Recent studies have suggested that mucosal surfaces, specifically the periodontium, the gut, and the lungs, might be privileged sites of autoimmunity initiation in RA [5] ignited by a humoral and cytotoxic immune response against mucosal-associated pathogens [6]. Accordingly, there is convincing evidence that immunity against mucosal microbial pathogens such as *Porphyromonas gingivalis* (*P. gingivalis)* and *Aggregatibacter *actinomycetemcomitans** (*A. actinomycetecomitans*) may be linked to RA development [7,8,9,10]. RA-related autoantibodies against these pathogens are stimulated in different ways. PPAD—an enzyme with citrullination activity against P.g—and LtxA—an enzyme with hyper citrullination activity via neutrophil osmotic lysis to A.a—are known as essential factors to sustain RA development [11,12,13].

Higher prevalence with respect to the general population of the humoral immune response against *Mycobacterium avium* subsp. *paratuberculosis* (MAP), Epstein–Barr virus (EBV), and human endogenous retrovirus (HERVs, ancient viruses integrated into the human genome) has also been demonstrated in patients with several immune-mediated states, including RA [14,15,16,17].

Among mechanisms potentially involved in microbial-driven RA pathogenesis, molecular mimicry, epitope spreading, and bystander activation are the most commonly reported [7].

A comprehensive demonstration of the role of the immune response against multiple pathogens in RA pathogenesis is still lacking. Therefore, this study aimed to evaluate the prevalence and magnitude of the immune response against different highly immunogen microbial peptides derived from *P. gingivalis* (RgpA, Kpg), *A. actinomycetecomitans* (LtxA-1, LtxA-2) MAP (MAP_4027_18–32_), EBV (EBNA1_400–413_, BOLF1_305–320_), and HERV-W env-su 93–108 in RA patients compared with the general population.

## 2. Materials and Methods

### 2.1. Subjects

Consecutive unselected RA patients attending the outpatient Rheumatology Unit at the Department of Clinical and Experimental Medicine of the University of Sassari (Italy) during the period between 2019 and 2020 and fulfilling the 2010 American College of Rheumatology classification criteria [18,19] were enrolled in the study. To evaluate the presence of correlations between humoral immune response and RA-specific features, the following disease-specific scores, disease descriptors, and treatment data were collected: current steroid treatment; cumulative dosage of steroids (last 12 months); current treatment with biological or targeted-synthetic disease-modifying anti-rheumatic drugs (b/tsDMARDs); current use of methotrexate; cumulative; C-reactive protein (CRP) concentrations, mg/dL; erythrocyte sedimentation rate (ESR), mm/h; Disease Activity Score-28 (DAS-28); Clinical Disease Activity Index (CDAI). In addition, as a control group, we enrolled age- and sex-matched healthy blood donors attending the local blood transfusion service.

The Ethics Committee of the University of Cagliari approved this study (PG 2018/5643). Informed consent was obtained from all individual participants.

### 2.2. Peptide Synthesis

Peptides derived from *Pg* (RgpA: ADPVVTTQNIIVT and Kgp: VTDLYYSAVDGD) and *Aa* (LtxA-1: AWENKYGKNTFENGYDA and LtxA-2: TALIKAAQKLGIEVYHE) were designed using the Immune Epitope Database and Analysis Resource (IEBD) and synthesized at >95% purity (LifeTein, South Plainfield, NJ, USA). Peptides derived from *EBV* (EBNA1_400–413_: PGRRPFFHPVGEAD), BOLF1_305–320_: AAVPVLAFDAARLRLLE), *MAP* (MAP_4027_18–32_: AVVPVLAYAAARLL), and *HERV-W* (HERV-W env-su _93–108_: NPSCPGGLGVTVCWTY) were selected from previous studies [20,21,22]. All peptides were dissolved in dimethyl sulfoxide (DMSO) and stored at −80 °C in single-use aliquots (10 mM).

### 2.3. Enzyme-Linked Immunosorbent Assay (ELISA)

Sera were separated according to the standard method [23]. Briefly, we allowed blood to clot for 30–60 min at room temperature. Then, we centrifugated it for 10 min at approximately 1000g, and aliquoted serum were preserved at –80-degree freezer. Indirect ELISA was carried out to detect specific Abs against the select antigens included in the study, as reported previously [24]. Briefly, 10 µg/mL of each peptide with a solution 0.05 M of carbonate–bicarbonate, pH 9.5 (Sigma-Aldrich, St. Louis, MO, USA) were coated in 96-well plates and incubated overnight at 4 ◦C. Plates were incubated for one hour at room temperature (RT) in a blocking solution with 5% non-fat dried milk (Sigma-Aldrich, St. Louis, MO, USA) and phosphate-buffered saline (PBS) (Sigma-Aldrich, St. Louis, MI, USA) and washed twice with 0.05% Tween-20–PBS 1X (PBS-T) (Sigma-Aldrich, St. Louis, MI, USA). Then, one microliter of serum samples was added to plates and incubated for two hours. Then, each plate was washed five times in PBS-T and incubated for one hour at RT with 100 µL of PBS and anti-human IgG polyclonal antibody (1:1000, Sigma-Aldrich, St. Louis, MI, USA). After another washing step in PBS-T, plates were incubated in a dark environment for eight to ten minutes in milli-Q water and p-nytrophenyl phosphate (Sigma-Aldrich, St. Louis, MI, USA), and the optical density was read at a wavelength of 405 nm using a microplate reader (Molecular Devices, Sunnyvale, CA, USA). All samples were repeated in triplicate, and positive controls were used for each peptide. The obtained absorbance values (at 405 nm) were normalized to a highly positive control serum with absorbance reactivity set at 1.0 arbitrary units (AU/mL). Results are expressed as means of triplicate 405 nm OD values.

Intra-assay variation was calculated based on the mean of the CV percentages (%CVs) obtained from OD measurements repeated three times for each serum in three-well plates. Inter-assay variation was calculated based on the mean of %CVs obtained from experiments repeated three times for each serum in three separate plates during three different days. Inter-assay variation was done for 40 serum samples with high, low, and moderate ODs.

### 2.4. Statistical Analysis

The results were expressed as a mean of three separate experiments, and the analysis was performed using GraphPad Prism version 8.0 software (San Diego, CA, USA).

A *T*-test and Fisher’s exact test were performed to analyze the matching age and sex in RA patients with HC group. The Mann–Whitney test was performed for non-parametric comparisons. A value of *p* < 0.05 was considered significant. The cut-off for positivity was established in the interval 0.35–0.52 (AU)/mL based on the receiver operating characteristic (ROC) curve with ≥90% specificity and 95% confidence interval. In addition, Fisher’s exact test was employed to compare the percentages of positive subjects in the two groups. The correlation between OD values obtained by the ELISA test from different peptides, and the RA features, RA activity (DAS-28), systemic inflammation (ESR, CRP), and type of immunosuppressive treatment was explored by bivariate correlation and regression analysis with Stata.

## 3. Results

This retrospective case-control study examined a set of serum samples derived from 148 RA patients (123 females, 25 males; median ± SD: 65.2 ± 9) and 148 healthy controls (120 females, 28 males; median ± SD: 63.5 ± 7). The demographic and clinical features of all subjects involved in the present study are summarized in Table 1.

No significant difference was observed between the age and sex of HC compared to RA patients’ groups (*p* > 0.077 and *p* > 0.76).

The variation was from 7.2% to 9.6% for intra-assay and from 10.1% to 13.7% for inter- assay.

**Figure 1:**
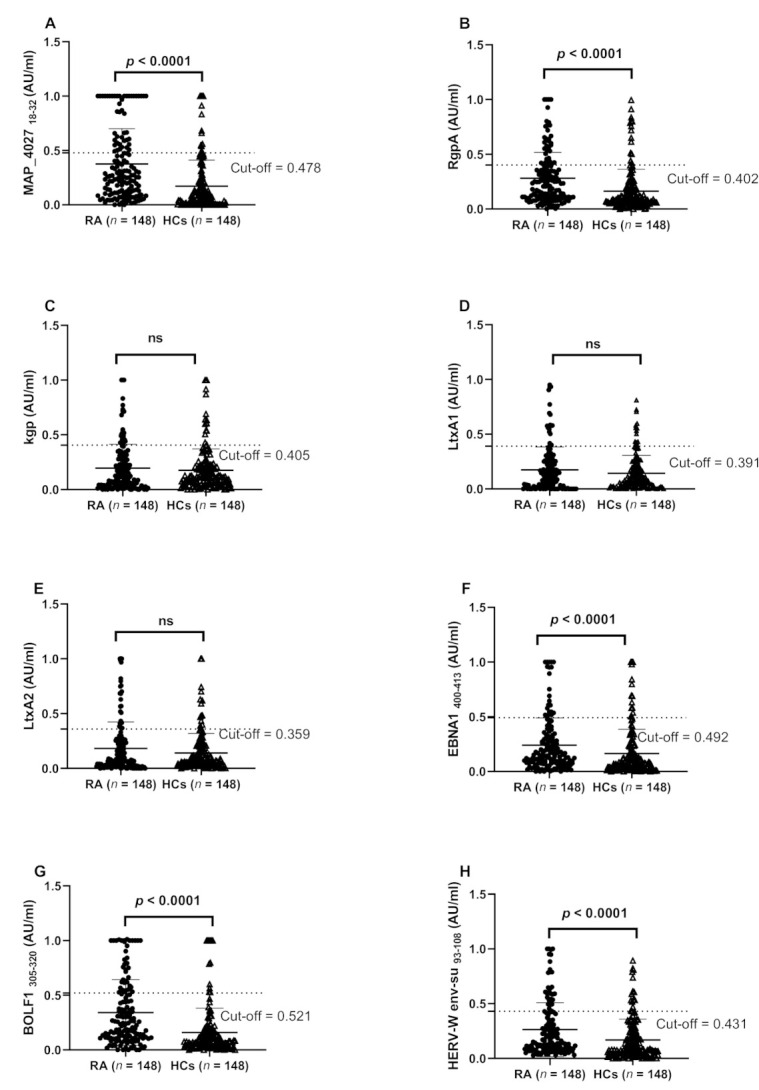
ELISA-based analysis of Abs reactivity against pathogenic microorganism-derived peptides in RA patients and HCs. Sera samples were tested against plate-coated (**A**) MAP4027, (**B**) RgpA, (**C**) Kpg, (**D**) LtxA1, (**E**) LtxA2, (**F**) EBNA1, (**G**) EBVBOLF, and (**H**) HERV-W env peptides. Dashed lines represent thresholds used to assess the samples’ positivity (cut-off value based on the ROC curve with ≥90% specificity and 95% confidence interval).

**Figure 2:**
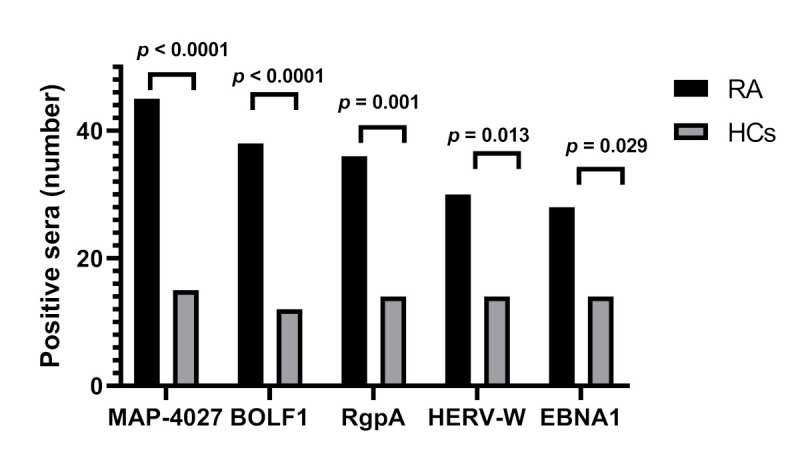
Number of sera positive to MAP4027, BOLF1, RgpA, HERV-W, and EBNA1 peptides in RA and HCs (Fisher’s exact test: *p* < 0.05).

Among the tested immunogen peptides, the highest titer of antibodies was demonstrated against the MAP4027 peptide, corresponding to a seroreactivity of 30.4% (*n* = 45) among RA patients and 10.1% (*n* = 15) in HCs (AUC = 0.736, *p* < 0.0001) (Figure 1A, Figure 2).

We also demonstrated a strong Ab response against both *P. gingivalis*-derived peptides in RA sera compared with HCs that reached statistical significance for the RgpA peptide (*p* < 0.0001) (Figure 1B,C). Therefore, 36 (24.3%) out of 148 RA sera and 14 (9.4%) out of 148 HCs sera were anti-RgpA positive (AUC = 0.705, *p* = 0.001) (Figure 1B, Figure 2). On the contrary, the humoral immune response against *A. actinomycetecomitans*-derived peptides was not significantly different between groups (Figure 1D,E).

Similarly, as expected, the titer and prevalence of Abs against EBV (EBNA1, BOLF1) was significantly higher in RA sera than in the counterpart (*p* < 0.0001) (Figure 1F,G). This corresponds to 38 (25.7%) and 28 (18.9%) of RA sera being positive for BOLF and EBNA1 compared with 12 (8.1%) and 14 (9.4%) of control sera, respectively (AUC = 0.647, and AUC = 0.736, respectively; *p* < 0.0001 and *p* = 0.029, respectively) (Figure 1F,G; Figure 2).

In addition, Abs titers against peptides derived from HERV-W (HERV-W env-su) were significantly higher in RA than in HCs (*p* < 0.0001) (Figure 1H). This figure corresponds to 20.3% (*n* = 30) of RA sera seropositivity against anti-HERV-W env-su compared with 9.4% (*n* = 14) of its counterpart (AUC = 0.736, *p* < 0.0001; *p* = 0.013) (Figure 1H, Figure 2).

In total, 53 (35.8%) out of 148 RA sera and 93 (62.8%) out of 148 HCs were negative for all tested peptides (*p* < 0.0001).

There was no significant difference between OD values in patients < 1 year disease duration compared with patients >1 year disease duration (*p* > 0.05).

**Figure 3:**
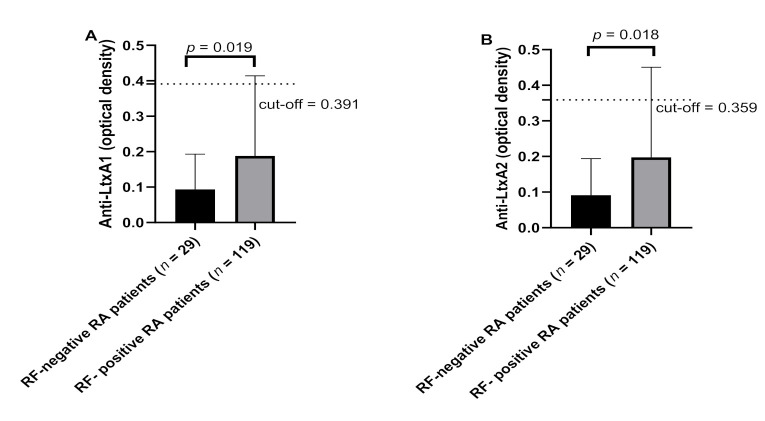
Abs response against LtxA1 (**A**) and LtxA2 (**B**) in RF-positive RA patients vs. RF-negative RA patients. The black bars represent the average ±S, dashed lines represent thresholds used to assess the samples’ positivity.

Of note, we found an increased titer and prevalence of antibodies against LtxA1 and LtxA2 in seropositive vs. seronegative RF among RA patients (Figure 3). The correlation analysis between remaining RA predictors and Abs was not significant (Table S1).

**Figure 4:**
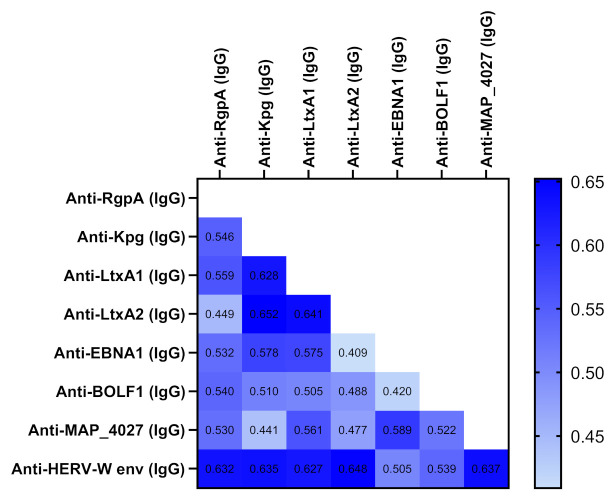
Heatmap displaying the *r* values obtained from Spearman correlation analysis performed among derived peptides.

Correlation analyses were performed according to OD values obtained by ELISA test for different peptides (Figure 4).

There was a significant correlation between all Abs (*p* < 0.05). Higher correlation was observed between anti-LtxA2 and anti-Kpg (*r* = 0.652, *p* < 0.0001) followed anti-HERV-W andanti-LtxA2 (*r* = 648, *p* < 0.0001), anti-LtxA1 and anti-LtxA2 (*r* = 641, *p* < 0.0001), anti-MAP4027 and anti-HERV-W (*r* = 0.637, *p* < 0.0001), anti-Kpg and anti-HERV-W (*r* = 0.635, *p* < 0.0001), anti-HERV-W and anti-RgpA (*r* = 0.632, *p* < 0.0001), anti-LtxA1 and anti-Kpg (*r* = 0.628, *p* < 0.0001), and anti-HERV and anti-LtxA1 (*r* = 0.627, *p* < 0.0001). The heatmap (Figure 4) shows the *r* values between pairs of epitopes.

## 4. Discussion

Several environmental factors, including infections, have been associated with an increased risk of RA [7,25,26].

We tested the humoral response against selected peptides derived from pathogens previously associated with RA, including *P. gingivalis*, *A. actinomycetemcomitans*, MAP, EBV, and HERV-W in RA patients in comparison to HCs. We found that the highest prevalence of humoral response was against MAP, suggesting a contributing role for this microorganism in RA development [8,10]. After colonization of MAP in the host, it can evade the immune system through different mechanisms such as molecular mimicry, which is a condition that may lead to the host immune system targeting self-epitopes [7].

This bacterium is the causative agent of paratuberculosis, which is a disease predominately found in ruminants that may spread to human hosts by water and foodborne transmission routes [27]. This pathogen is associated with Crohn’s disease and other autoimmune diseases in humans [27]. The potential role of MAP in RA has been already highlighted in a previous study in which RA sera showed a remarkably frequency of reactivity against PtpA (*p* < 0.001) and PknG (*p* = 0.0054) peptides in comparison to HCs [10]. A significant linear correlation between the number of swollen joints and the concentrations of antibodies against PtpA was also found (*p* = 0.018) [10]. Accordingly, we previously demonstrated significant cross-reactivity between MAP (MAP4027) and interferon regulatory factor 5 peptide (IRF5_424–434_) [20] in RA.

In this study, the Ab response against peptides derived from lysogenic phase (BOLF1) and latent phase (EBNA1) proteins of EBV was significantly higher in RA patients compared with the control group. This result support previous evidence regarding the role of EBV in RA [28,29]. Of note, EBV is potentially involved in the activation and stimulation of HERV-K expression [30,31,32]. For the first time, we found a strong reactivity in RA against the selected surface epitope of HERV-W (HERV-W env-su _93–108_). These results are in line with other studies reporting increased humoral responses to EBV and HERV-K peptides in Sardinian patients with different autoimmune diseases, including RA [28,33]. Interestingly, our results have shown high correlation between the HERV-W env peptide and LTX2, MAP4027, Kpg, and RgpA, which probably supports the hypothesis that these pathogens might act synergically to induce autoimmunity through a common target.

Furthermore, we found that RA compared with HCs show a higher prevalence of humoral response against peptides derived by periodontal pathogens, which was statistically meaningful for the anti-RgpA IgG peptide. This is in line with findings from epidemiological studies suggesting a potential pathogenic link between periodontitis and RA [11,34,35]. *P. gingivalis* and *A. actinomycetecomitans* are the most common reported pathogens in periodontitis, and they can contribute to RA autoantibody production through various mechanisms: directly by post-translation modification of human protein (by the PPAD enzyme of *P. gingivalis*) or indirectly by neutrophil osmotic lysis (leukotoxin of Aa) [9,11,12,13]. In this study, we found a positive significant correlation between anti-LtxA2 and anti-Kpg, and also, anti-LtxA1 and anti-LtxA2 with RF, suggesting that *P. gingivalis* and *A.* *actinomycetecomitans* may cooperate in inducing immunity against periodontal and synovial self-antigens. Although data from in vitro and in vivo studies on the interaction between these two pathogens are scarce, co-infection seems to be associated with poor RA prognosis [36]. One potential limitation of the current study is the absence of RA patients in the preclinical period for the evaluation of antibodies against our peptides. Moreover, a lack of anti-CCP titer and its quantitative evaluation among RA patients is another limitation of this investigation.

In conclusion, this study demonstrated a link between different pathogens and RA. The exposure to these pathogens, either in the preclinical period (before the disease onset) or during the clinical phase, is likely to have a pivotal role in the emergence and maintenance of RA. Further investigations are needed to confirm these results in larger groups of RA patients.

## Figures and Tables

**Table 1:** Demographic and clinical characteristics of patients and healthy controls tested.

	Rheumatoid Arthritis *n* = 148	Healthy Controls *n* = 148	*p* Value
Age, years	65.2	63.5	*p* > 0.077
Female, *n* (%)	123 (83.1)	120 (81)	*p* > 0.76
Disease duration, months	108 (8.2)	/	
ACPA, (%)	74.2	/	
RF, (%)	81.0	/	
DAS28	3.9 (0.1)	/	
CDAI	10.9 (0.7)	/	
CRP, mg/L	3.7 (2.4)	/	
ESR, mm/h	32.3 (1.8)	/	
Steroid use, *n* (%)	37.1	/	
DMARDs use, (%)	70.2	/	
Methotrexate use, (%)	55.7	/	
TNFi use, (%)	23.4	/	
Abatacept use, (%)	4.7	/	
Tocilizumab use, (%)	5.4	/	

Values are mean and proportions. DAS28, Disease Activity Score-28; CDAI, clinical disease activity index; ACPA, anti-citrullinated peptide antibodies; RF, rheumatoid factor; CRP, C-reactive protein concentrations, mg/dL; ESR, erythrocyte sedimentation rate, mm/h; DMARDs, synthetic disease-modifying anti-rheumatic drugs; TNFi, tumor necrosis factor inhibitors.

## Data Availability

The data that support the findings of this study are available from the corresponding author, upon reasonable request.

## Supplementary Materials

The following are available online at https://www.mdpi.com/article/10.3390/jcm10215153/s1, Table S1: Spearman correlation analysis (*r* and *p* values) performed between OD values obtained by ELISA test and RA features.
