# Supplementary material for: Humoral Response to Microbial Biomarkers in Rheumatoid Arthritis Patients
Source: J Clin Med. 2021 Nov 2;10(21):5153. doi: 10.3390/jcm10215153 (PMC8584451; doi:10.3390/jcm10215153)
Supplement: Supplementary file 1 [file jcm-10-05153-s001.zip › jcm-1423193-supplementary.pdf]

Table S1. Spearman correlation analysis (r and *p* values) performed between OD values obtained by ELISA test and RA features.

	Anti-MAP4027	Anti-MAP4027*	Anti-RgpA	Anti-RgpA*	Anti-Kpg	Anti-Kpg*	Anti-LtxA1	Anti-LtxA1*	Anti-LtxA2	Anti-LtxA2*	Anti-EBNA1	Anti-EBNA1*	Anti-BOLF	Anti-BOLF*	Anti-HERVW	Anti-HERVW*
Age	−0.203	−0.0202	−0.092	−0.021	0.087	0.121	0.147	0.110	0.202	0.131	−0.172	−0.147	0.116	0.170	−0.165	−0.082
	0.064	0.065	0.268	0.794	0.297	0.146	0.079	0.186	0.095	0.115	0.088	0.077	0.165	0.050	0.057	0.328
Sex	−0.057	−0.094	−0.074	−0.080	−0.027	−0.065	0.017	0.057	0.014	0.051	−0.140	−0.150	−0.124	−0.106	−0.005	0.003
	0.484	0.255	0.367	0.329	0.741	0.432	0.832	0.488	0.861	0.533	0.089	0.067	0.131	0.197	0.946	0.970
Disease duration	0.0001	0.056	0.094	0.029	0.134	0.064	0.170	0.106	0.124	0.137	−0.015	−0.053	0.074	0.061	−0.002	0.001
	0.999	0.494	0.253	0.720	0.103	0.441	0.039	0.201	0.132	0.096	0.856	0.523	0.372	0.459	0.977	0.988
ACPA	−0.058	−0.026	−0.055	−0.020	0.046	0.019	0.068	0.112	0.097	0.142	−0.108	−0.140	0.028	0.030	−0.046	−0.033
	0.503	0.764	0.524	0.819	0.597	0.825	0.435	0.198	0.266	0.102	0.214	0.108	0.743	0.732	0.599	0.703
RF	0.065	0.041	0.0700	0.064	0.125	0.104	0.178	0.177	0.178	0.149	−0.002	−0.020	0.108	0.021	0.079	0.062
	0.447	0.630	0.417	0.453	0.146	0.227	0.038	0.038	0.037	0.083	0.978	0.815	0.206	0.803	0.355	0.469
DAS28	0.087	0.114	0.020	0.030	−0.043	−0.094	0.019	0.011	−0.068	−0.042	0.008	0.052	−0.091	−0.085	0.131	0.153
	0.301	0.177	0.807	0.717	0.606	0.266	0.818	0.894	0.422	0.614	0.921	0.533	0.279	0.316	0.119	0.069
CDAI	0.072	0.143	−0.048	−0.006	−0.102	−0.156	−0.075	−0.112	−0.090	−0.028	0.011	0.092	−0.133	−0.0108	0.058	0.059
	0.385	0.083	0.558	0.934	0.215	0.058	0.366	0.173	0.276	0.734	0.888	0.265	0.106	0.189	0.485	0.471
ESR	0.078	0.039	0.055	0.010	−0.045	−0.088	0.0930	0.0929	−0.055	−0.024	0.063	0.015	0.013	−0.011	0.117	0.183
	0.355	0.636	0.514	0.904	0.587	0.292	0.272	0.271	0.508	0.770	0.451	0.858	0.869	0.890	0.162	0.029
CRP	−0.056	−0.071	0.008	−0.062	−0.004	−0.042	−0.055	−0.038	−0.072	−0.045	−0.033	−0.050	−0.008	−0.013	0.090	0.145
	0.504	0.395	0.916	0.457	0.960	0.611	0.512	0.651	0.386	0.589	0.689	0.547	0.924	0.870	0.278	0.082
Steroid	0.067	0.038	0.069	−0.012	0.035	0.071	0.122	0.056	0.110	0.041	0.032	0.056	−0.122	−0.099	0.057	−0.040
	0.412	0.639	0.400	0.881	0.667	0.386	0.140	0.498	0.180	0.620	0.691	0.491	0.136	0.227	0.491	0.629
DMARD	−0.031	−0.020	−0.000	−0.044	0.108	0.0640	0.117	0.151	0.016	0.005	0.013	−0.025	0.060	0.145	−0.016	−0.003
	0.705	0.809	0.998	0.589	0.189	0.439	0.156	0.065	0.843	0.947	0.870	0.758	0.465	0.077	0.840	0.971
Methotrexate	0.009	−0.032	0.060	0.029	0.091	0.066	0.105	0.081	0.093	0.059	0.143	0.083	0.123	0.212	0.081	0.111
	0.909	0.693	0.466	0.725	0.270	0.424	0.203	0.324	0.258	0.472	0.083	0.317	0.135	0.009	0.325	0.180
TNFi	0.018	−0.011	0.123	0.139	0.0904	0.133	0.034	0.042	0.043	0.108	0.105	0.064	0.154	0.083	0.074	0.084
	0.821	0.886	0.134	0.090	0.274	0.107	0.677	0.608	0.602	0.189	0.201	0.437	0.061	0.313	0.365	0.308
Abatacept	−0.076	−0.078	−0.063	−0.052	−0.051	−0.003	−0.043	−0.082	−0.107	−0.0980	−0.085	−0.107	−0.076	−0.058	−0.069	−0.033
	0.353	0.345	0.441	0.529	0.532	0.965	0.598	0.316	0.193	0.235	0.299	0.192	0.356	0.483	0.400	0.689
Tocilizumab	0.008	0.036	0.075	0.143	−0.079	−0.0159	−0.008	0.002	0.003	−0.024	0.071	0.113	−0.050	−0.072	0.061	−0.046
	0.921	0.656	0.361	0.082	0.339	0.848	0.917	0.976	0.963	0.771	0.386	0.169	0.546	0.383	0.495	0.577

r and *p* values obtained from Spearman correlation analysis performed between OD values obtained by ELISA test for different peptides and RA features. Significant values are marked in red. ACPA, anti-citrullinated peptide antibodies; RF, rheumatoid factor; DAS28, disease activity score 28 joints; CDAI, clinical disease activity index; ESR, erythrocyte sedimentation rate, mm/h; CRP, C-reactive protein concentrations, mg/dL; DMARDs, synthetic disease-modifying anti-rheumatic drugs; TNFi, tumor necrosis factor inhibitors. *, OD values above cut-off values (Positive for peptide).
